# Structured reporting of computed tomography in the polytrauma patient assessment: a Delphi consensus proposal

**DOI:** 10.1007/s11547-023-01596-8

**Published:** 2023-01-19

**Authors:** Vincenza Granata, Roberta Fusco, Diletta Cozzi, Ginevra Danti, Lorenzo Faggioni, Duccio Buccicardi, Roberto Prost, Riccardo Ferrari, Margherita Trinci, Michele Galluzzo, Francesca Iacobellis, Mariano Scaglione, Michele Tonerini, Francesca Coppola, Chandra Bortolotto, Damiano Caruso, Eleonora Ciaghi, Michela Gabelloni, Marco Rengo, Giuliana Giacobbe, Francesca Grassi, Luigia Romano, Antonio Pinto, Ferdinando Caranci, Elena Bertelli, Paolo D’Andrea, Emanuele Neri, Andrea Giovagnoni, Roberto Grassi, Vittorio Miele

**Affiliations:** 1 Division of Radiology, Istituto Nazionale Tumori IRCCS Fondazione Pascale - IRCCS Di Napoli, Naples, Italy; 2 Medical Oncology Division, Igea SpA, Naples, Italy; 3 Division of Radiology, Azienda Ospedaliera Universitaria Careggi, Florence, Italy; 4 Italian Society of Medical and Interventional Radiology SIRM, SIRM Foundation, Milan, Italy; 5 Academic Radiology, Department of Translational Research, University of Pisa, Via Roma, 67, 56126 Pisa, Italy; 6 Imaging Department, San Paolo Hospital, Savona, Italy; 7 Radiology Unit, Azienda Ospedaliera Brotzu, Cagliari, Italy; 8 Department of Emergency Radiology, Azienda Ospedaliera San Camillo-Forlanini, Rome, Italy; 9 Department of General and Emergency Radiology, “A. Cardarelli” Hospital, Naples, Italy; 10 Department of Clinical and Experimental Medicine, University of Sassari, Sassari, Italy; 11 Department of Emergency Radiology, University Hospital of Pisa, Pisa, Italy; 12 Department of Radiology, AUSL Romagna, Faenza, Italy; 13 Department of Radiology, I.R.C.C.S. Policlinico San Matteo Foundation, Pavia, Italy; 14 Department of Medical Surgical Sciences and Translational Medicine, Sapienza University of Rome, Rome, Italy; 15 Research and Development, Exprivia Spa, Bari, Italy; 16 Department of Radiological Sciences, Oncology and Anatomical Pathology, Sapienza University of Rome, Rome, Italy; 17 Division of Radiology, Università Degli Studi Della Campania “Luigi Vanvitelli”, Naples, Italy; 18 Radiology Unit, C.T.O. Hospital, Azienda Ospedaliera Dei Colli, Naples, Italy; 19 Radiology Unit, “Sant’Anna E San Sebastiano” Hospital, Caserta, Italy; 20 Department of Clinical, Special and Dental Sciences, Università Politecnica Delle Marche, Ancona, Italy; 21 Department of Radiology, University Hospital “Azienda Ospedaliera Universitaria delle Marche”, Ancona, Italy

**Keywords:** Radiology report, Structured report, Polytrauma, Computed tomography

## Abstract

**Objectives:**

To develop a structured reporting (SR) template for whole-body CT examinations of polytrauma patients, based on the consensus of a panel of emergency radiology experts from the Italian Society of Medical and Interventional Radiology.

**Methods:**

A multi-round Delphi method was used to quantify inter-panelist agreement for all SR sections. Internal consistency for each section and quality analysis in terms of average inter-item correlation were evaluated by means of the Cronbach’s alpha (C*α*) correlation coefficient.

**Results:**

The final SR form included 118 items (6 in the “Patient Clinical Data” section, 4 in the “Clinical Evaluation” section, 9 in the “Imaging Protocol” section, and 99 in the “Report” section). The experts’ overall mean score and sum of scores were 4.77 (range 1–5) and 257.56 (range 206–270) in the first Delphi round, and 4.96 (range 4–5) and 208.44 (range 200–210) in the second round, respectively.

In the second Delphi round, the experts’ overall mean score was higher than in the first round, and standard deviation was lower (3.11 in the second round vs 19.71 in the first round), reflecting a higher expert agreement in the second round. Moreover, C*α* was higher in the second round than in the first round (0.97 vs 0.87).

**Conclusions:**

Our SR template for whole-body CT examinations of polytrauma patients is based on a strong agreement among panel experts in emergency radiology and could improve communication between radiologists and the trauma team.

**Supplementary Information:**

The online version contains supplementary material available at 10.1007/s11547-023-01596-8.

## Introduction

Trauma is the main cause of death for patients younger than 45 years old in Western Countries [1, 2], with a considerable proportion of all polytrauma due to car crashes. The number of casualties continues to rise, and according to the World Health Organization, every year the lives of approximately 1.3 million people are cut short because of a road traffic crash [3].

The optimal management of polytrauma patients requires a national trauma organization, which should define every level of the system, including all units of the trauma team [3, 4].

The suitable management of polytrauma patients can be challenging, and an efficient treatment requires that it begins at the accident site and should be maintained in all treatment phases. Therefore, treatment should be operated by a multidisciplinary team directed by a trauma surgeon to adequately manage severe injuries [4]. The multidisciplinary team should recognize as soon as possible life-threatening injuries or hemodynamically unstable patients according to vital parameters and classify them as polytrauma or non-polytrauma [5].

A decisive feature in the evolution of polytraumatized patient treatment is the possibility to obtain a rapid diagnosis, thanks to the opportunity to perform a whole-body computed tomography (CT) examination [5–7]. However, regarding which diagnostic tool to employ in polytrauma patients, the tenth edition of the Advanced Trauma Life Support (ATLS) suggests CT only if indicated, not by default, and only to assess specific body regions [8]. In contrast, the newest guidelines of the European Society of Emergency Radiology (ESER) endorse the approach proposed by ATLS in non-polytrauma patients and suggest whole-body CT as the diagnostic tool to use in polytrauma patients [9].

Currently, the optimal CT study protocol for polytrauma is a matter of debate [10–18]. Another critical issue is an efficient communication of imaging data to the trauma team, given the need to report trauma-related injuries as soon as possible [19–25]. The tool utilized to communicate radiological data to the trauma team is the radiological report. Traditionally, radiology reports are free text reports (FTR) based on descriptive communication, which could lead to confusion or even improper patient management if the report is unclear and difficult to understand for style and content [26–32]. This can be especially true in the emergency setting, where time is crucial and life-threatening conditions are usually dealt with, highlighting the need to organize FTR into structured reports (SR) [33–36]. SR allows to employ a checklist where all relevant items for a particular disease are reported, to avoid missing crucial data [37–44]. This structure can improve communication with referring clinicians by favoring quality and standardization of radiological reports. In this context, several radiological societies [including the Radiological Society of North America (RSNA) and the Italian Society of Medical and Interventional Radiology (SIRM)] have promoted the use and diffusion of SR in clinical practice [45, 46].

The aim of our study is to develop a SR template for whole-body CT imaging of polytrauma patients.

## Methods

### Expert panel

We performed a multi-round consensus-building Delphi exercise to create a complete SR template based on whole-body CT examinations for the assessment of polytrauma patients.

Following extensive debate within a working team of seven SIRM radiologist members experienced in emergency radiology, a first draft of the SR template for the reporting of polytrauma CT examinations was created.

A panel of 18 experts was set up, including members from the Italian College of Emergency Radiologists and the Imaging Informatics Chapter of SIRM. The panelists revised the initial draft iteratively to obtain a final consensus on SR.

### Selection of the Delphi domains and items

The panelists evaluated literature data on the main scientific databases, including PubMed, Scopus and Google Scholar, to assess papers published from December 2000 to June 2022 on: (*a*) polytrauma, (*b*) CT protocols in emergency settings, and (*c*) Structured Radiology Reports. In addition, they assessed the latest guidelines on the management of traumatized patients and on trauma classification by major scientific societies [47, 48]. Each panelist developed and shared the list of Delphi items via emails and/or teleconferences.

The working group divided the SR into four sections: (*a*) Patient Clinical Data, (*b*) Clinical Evaluation, (*c*) Imaging Protocol and (*d*) Report. The Report section included the template and a sub-section, where it was possible to add the most significant (key) images.

Two Delphi rounds were carried out. During the first round, each panelist independently contributed to refining the SR draft by means of online meetings or email exchanges. The level of panelists’ agreement for each SR model was tested in the second Delphi through a Google Forms^®^ questionnaire shared by email. Each expert expressed individual comments for each specific template section by using a five-point Likert scale (1 = strongly disagree, 2 = slightly disagree, 3 = slightly agree, 4 = generally agree, 5 = strongly agree).

After the second Delphi round, the last version of the SR was generated on the dedicated RSNA website (www.radreport.org) by using a T-Rex template format, in line with IHE (Integrating Healthcare Enterprise) and the MRRT (Management of Radiology Report Templates) profiles, accessible as open source software, with the technical support of Exprivia^®^ (Exprivia SpA, Bari, Italy). These determine both the format of radiology report templates using version 5 of Hypertext Markup Language (HTML5) and the transporting mechanism to request, retrieve and stock these schedules. The radiology report was structured by using a series of “codified queries” integrated in the preselected sections of the T-Rex editor [49].

### Statistical analysis

The responses of each panel member were exported in Microsoft Excel^®^ format to facilitate data collection and statistical analysis.

All panel member ratings for each section were analyzed with descriptive statistics measuring the mean score, standard deviation, and sum of scores. An average score of 3 was considered good and a score of 4 excellent.

To measure the internal consistency of panel member ratings for each section of the report, a quality analysis based on the mean correlation between items with Cronbach's correlation coefficient alpha (C*α*) was performed [50, 51]. C*α* provides a measure of the internal consistency of a test or scale and is expressed as a number between 0 and 1. Internal consistency describes the extent to which all items in a test measure the same concept. C*α* was determined after each round.

The closer the coefficient C*α* to 1.0, the greater the internal consistency of the elements of the scale. A coefficient alpha (*α*) > 0.9 was considered excellent, *α* > 0.8 good, *α* > 0.7 acceptable, *α* > 0.6 questionable, *α* > 0.5 poor, and *α* < 0.5 unacceptable. However, in the iterations an *α* of 0.8 was considered a reasonable target for internal reliability.

Data analysis was performed using Matlab Statistics Toolbox^®^ (The MathWorks Inc., Natick, MA, USA).

## Results

### Structured report

The final SR form (Appendix 1) included 118 items, of which 6 in the “Patient Clinical Data” section, 4 in the “Clinical Evaluation” section, 9 in the “Imaging Protocol” section, and 99 in the “Report” section.

The “Patient Clinical Data” section should be automatically imported from the HIS/RIS and includes anthropometric data (such as weight, height, BMI, BSA, and age class).

The “Clinical Evaluation” section should also be automatically imported from the HIS/RIS and includes data on the type of trauma (such as road trauma, precipitation, burn). In addition, this section comprises data on pathophysiological criteria (e.g., GCS < 13, systolic arterial pressure < 90 mmHg, respiratory rate > 29/min, oxygen saturation in room air < 90%) and anatomical criteria (e.g., sensory/motor deficit, chest trauma with mobile parietal flap, penetrating injury, proximal fracture of 2 or more long bones, amputation proximal to elbow or knee).

The “Imaging Protocol” section consists of data on CT brand and model, contrast study protocol (including data on post-contrast acquisitions), contrast medium (active principle, commercial name, volume, flow rate, iodine concentration), and ongoing adverse events.

The “Report” section was created for vascular, thorax and abdominal lesions, according to the latest version of the American Association for the Surgery of Trauma (AAST) injury scoring scales [47], with the goal to provide an injury grade that reflects severity, guides management and aids in prognosis assessment. Regarding spinal trauma, the AO Spine Thoracolumbar and Subaxial Injury Classification systems [48], which are the result of a systematic assessment and revision of the Magerl classification [52], were used. This section includes all possible trauma-related findings and the injury grade. In addition, data on the type of vascular trauma (dissection, thrombosis, pseudoaneurysm, active bleeding) were included, as well as data on pleural effusion, pneumothorax, pneumomediastinum, pericardial effusion, peritoneal effusion, pneumoperitoneum, and other findings (fat embolism, foreign bodies, or incidental trauma-unrelated findings).

### Consensus agreement

Table 1 reports the single scores and the sum of scores of panel experts for the SR in the first Delphi round, whereas Table 2 reports their single scores and sum of scores for the SR in the second round.

**Table 1:** Single scores and sum of scores of SR panelists (first Delphi round)

Panelist #	1	2	3	4	5	6	7	8	9	10	11	12	13	14	15	16	17	18	Mean	SD
A1. Anthropometric data	5	5	5	5	3	5	5	5	5	5	5	2	3	5	5	5	5	4	4.56	0.92
B1. Clinical information—type of trauma	4	5	5	5	5	5	3	5	5	5	5	5	3	5	5	5	5	5	4.72	0.67
B2. Clinical information—pathophysiological criteria	4	5	5	5	2	5	2	5	5	4	5	4	2	2	2	5	5	5	4.00	1.33
B3. Clinical information—anatomical criteria	4	5	5	5	3	5	1	5	5	3	5	3	2	2	2	5	5	5	3.89	1.41
C1. Examination data	5	5	5	5	5	5	5	5	5	5	5	5	5	5	5	5	5	4	4.94	0.24
C2. Contrast medium	5	5	5	5	5	5	5	5	5	5	5	5	5	5	5	5	5	5	5.00	0.00
C3. Adverse events	5	5	5	5	5	5	3	5	5	5	5	5	5	5	5	5	5	5	4.89	0.47
D1. Cranial theca	5	5	5	5	5	5	3	3	5	5	5	5	5	5	5	5	5	4	4.72	0.67
D2. Extra-axial spaces	5	5	5	5	5	5	5	3	5	5	5	5	5	5	5	5	5	5	4.89	0.47
D3. Brain tissue	5	5	5	5	5	5	3	3	5	5	5	5	5	3	3	5	5	5	4.56	0.86
D4. Cerebellum and brainstem	5	5	5	5	5	5	4	3	5	5	5	5	5	3	3	5	5	5	4.61	0.78
D5. Midline	5	5	5	5	5	5	5	5	5	5	5	5	5	5	5	5	5	5	5.00	0.00
D6. Ventricular system	5	5	5	5	5	5	4	3	5	5	5	5	5	5	5	5	5	5	4.83	0.51
D7. Associated findings	5	5	5	5	5	5	5	5	5	5	5	5	5	5	5	5	5	5	5.00	0.00
E1. Vascular cervical injury	5	5	5	5	5	5	5	5	5	5	5	5	5	4	4	5	5	5	4.89	0.32
F1. Pleuro-pericardial effusion, pneumothorax/pneumomediastinum, tracheobronchial shaft lesions	5	5	5	5	5	5	5	5	5	5	5	5	5	4	4	4	5	5	4.83	0.38
F2. Chest wall injury	5	5	5	5	5	5	5	5	5	5	4	5	5	3	3	5	5	5	4.72	0.67
F3. Cardiac injury	4	5	5	5	5	5	2	5	5	5	5	5	4	2	2	5	5	5	4.39	1.14
F4. Lung injury	5	5	5	5	5	5	5	5	5	5	4	5	5	4	4	5	5	5	4.83	0.38
F5. Thoracic vascular injury	5	5	5	5	5	5	5	5	5	5	5	5	5	3	3	5	5	5	4.78	0.65
F6. Esophageal injury	5	5	5	5	5	5	5	5	5	5	5	5	5	3	3	5	5	5	4.78	0.65
F7. Diaphragmatic lesion	5	5	5	5	5	5	5	5	5	5	4	5	5	3	3	5	5	5	4.72	0.67
G1. Peritoneal effusion, pneumoperitoneum	5	5	5	5	5	5	5	5	5	5	5	5	5	4	4	4	5	5	4.83	0.38
G2. Abdominal vascular injury	5	5	5	5	5	5	5	5	5	5	5	5	5	3	3	5	5	5	4.78	0.65
G3. Splenic injury	5	5	5	5	5	5	5	5	5	5	4	5	5	4	4	5	5	5	4.83	0.38
G4. Liver injury Peripheral vascular injury I4. Other findings and conclusions J1. Meaningful key images	5	5	5	5	5	5	5	5	5	5	4	5	5	4	4	5	5	5	4.83	0.38
G5. Extrahepatic biliary lesion	5	5	5	5	5	5	5	5	5	4	5	5	5	3	3	5	5	5	4.72	0.67
G6. Pancreatic lesion	5	5	5	5	5	5	5	5	5	5	4	5	5	4	3	5	5	5	4.78	0.55
G7. Gastric lesion	5	5	5	5	5	5	5	5	5	5	5	5	5	3	3	5	5	5	4.78	0.65
G8. Duodenal injury	5	5	5	5	5	5	5	5	5	5	5	5	5	4	4	5	5	5	4.89	0.32
G9. Small intestine injury	5	5	5	5	5	5	5	5	5	5	5	5	5	4	4	5	5	5	4.89	0.32
G10. Colonic lesion	5	5	5	5	5	5	5	5	5	5	5	5	5	4	4	5	5	5	4.89	0.32
G11. Rectal injury	5	5	5	5	5	5	5	5	5	5	5	5	5	4	4	5	5	5	4.89	0.32
G12. Adrenal gland injury	5	5	5	5	5	5	4	5	5	5	4	5	5	3	3	5	5	5	4.67	0.69
G13. Kidney injury	5	5	5	5	5	5	5	5	5	5	0	5	5	3	3	5	5	5	4.50	1.29
G14. ureteral injury	5	5	5	5	5	5	5	5	5	5	5	5	5	3	3	5	5	5	4.78	0.65
G15. Bladder injury	5	5	5	5	5	5	5	5	5	5	5	5	5	4	4	5	5	5	4.89	0.32
G16. Urethral injury	4	5	5	5	5	5	2	5	5	4	5	5	5	3	3	4	5	5	4.44	0.92
G17. Uterine lesion (non-pregnant patient)	5	5	5	5	5	5	5	5	5	5	5	5	5	4	4	5	5	5	4.89	0.32
G18. Uterine lesion (pregnant patient)	5	5	5	5	5	5	5	5	5	5	5	5	5	4	4	5	5	5	4.89	0.32
G19. Fallopian tube injury	5	5	5	5	5	5	5	5	5	5	5	5	5	2	2	5	5	5	4.67	0.97
G20. Ovarian lesion	5	5	5	5	5	5	5	5	5	5	5	5	5	3	3	5	5	5	4.78	0.65
G21. Vaginal injury	4	5	5	5	5	5	5	5	5	5	5	5	5	3	3	5	5	5	4.72	0.67
G22. Vulvar lesion	4	5	5	5	5	5	5	5	5	4	5	4	4	5	5	5	5	5	4.78	0.43
G23. Testicular lesion	5	5	5	5	5	5	5	5	5	5	5	3	4	4	4	5	5	5	4.72	0.57
G24. Scrotal injury	5	5	5	5	5	5	5	5	5	5	5	3	4	4	4	5	5	5	4.72	0.57
G25. Penile lesion	5	5	5	5	5	5	5	5	5	5	5	3	4	4	4	5	5	5	4.72	0.57
H1. Cervical spine	5	5	5	5	5	5	5	5	5	5	5	5	5	4	4	5	5	5	4.89	0.32
H2. Dorso-lumbar spine	5	5	5	5	5	5	5	5	5	5	5	5	5	4	4	5	5	5	4.89	0.32
H3. Sacro-coccyx	5	5	5	5	5	5	4	5	5	5	5	5	5	5	5	5	5	5	4.94	0.24
H4. Pelvic girdle	5	5	5	5	5	5	5	5	5	5	5	5	5	5	5	4	5	5	4.94	0.24
I1. Upper limb fracture	5	5	5	5	5	5	5	5	5	5	5	5	5	5	5	5	5	5	5.00	0.00
I2. Fracture of the lower limb	5	5	5	5	5	5	5	5	5	5	5	5	5	5	5	5	5	5	5.00	0.00
I3. Peripheral vascular injury	5	5	5	5	5	5	5	5	5	5	5	5	5	3	3	5	5	5	4.78	0.65
Sum of scores	263	270	270	270	263	270	245	260	270	264	258	257	255	207	206	266	270	267	257.28	19.66

**Table 2:** Single scores and sum of scores of SR panelists (second Delphi round)

Panelist #	1	2	3	4	5	6	7	8	9	10	11	12	13	14	15	16	17	18	Mean	SD
A1. Anthropometric data	5	5	5	4	5	5	5	5	5	5	5	5	5	5	5	5	5	5	4.94	0.24
B1. Clinical information—type of trauma	5	5	5	5	5	5	5	5	4	5	5	5	5	5	5	5	5	5	4.94	0.24
B2. Clinical information—pathophysiological criteria	5	5	5	5	5	5	5	5	5	5	5	5	5	5	5	5	5	5	5.00	0.00
B3. Clinical Information—Anatomical Criteria on Physical Exam	5	5	5	5	5	5	5	5	5	5	5	5	5	5	5	5	5	5	5.00	0.00
C1. Examination data	5	5	5	5	5	4	5	5	5	5	5	5	5	5	5	5	5	5	4.94	0.24
C2. Contrast medium	5	5	5	5	5	4	5	5	5	5	5	5	5	5	5	5	5	5	4.94	0.24
C3. Adverse events	5	5	5	5	5	5	5	5	5	5	5	5	5	5	5	5	5	5	5.00	0.00
D1. Cranial theca	5	4	5	5	5	4	5	5	5	5	5	5	5	5	5	5	5	5	4.89	0.32
D2. Extra-axial spaces	5	4	5	5	5	5	5	5	5	5	5	5	5	5	5	5	5	5	4.94	0.24
D3. Brain tissue	5	4	5	5	5	5	5	5	5	5	5	5	5	5	5	5	5	5	4.94	0.24
D4. Midline	5	5	5	5	5	5	5	5	5	5	5	5	5	5	5	5	5	5	5.00	0.00
D5. Ventricular system	5	5	5	5	5	5	5	5	5	5	5	5	5	5	5	5	5	5	5.00	0.00
D6. Associated findings	5	5	5	5	5	4	5	5	5	5	5	5	5	5	5	5	5	5	4.94	0.24
E1. Vascular cervical injury	5	5	5	5	5	5	5	5	5	5	5	5	5	5	4	4	5	5	4.89	0.32
F1. Pleuro-pericardial effusion, pneumothorax/pneumomediastinum, tracheobronchial shaft lesions	5	5	5	5	5	5	5	5	5	5	5	5	5	5	5	5	5	5	5.00	0.00
F2. Chest wall injury	5	5	5	5	5	5	5	5	5	5	5	5	5	5	5	4	5	5	4.94	0.24
F3. Cardiac injury	5	5	5	5	5	5	5	5	5	5	5	5	5	5	5	5	5	5	5.00	0.00
F4. Lung injury	5	5	5	5	5	4	5	5	5	5	5	5	5	5	5	4	5	5	4.89	0.32
F5. Thoracic vascular injury	5	5	5	5	5	4	5	5	5	5	5	5	5	5	4	4	5	5	4.83	0.38
F6. Esophageal injury	5	5	5	5	5	4	5	5	5	5	5	5	5	5	5	5	5	5	4.94	0.24
F7. Diaphragmatic lesion	5	5	5	5	5	5	5	5	5	5	5	5	5	5	5	5	5	5	5.00	0.00
G1. Peritoneal effusion, pneumoperitoneum	5	5	5	5	5	5	5	5	5	5	5	5	5	5	5	4	5	5	4.94	0.24
G2. Abdominal vascular injury	5	5	5	5	5	5	5	5	5	5	5	5	5	5	4	4	5	5	4.89	0.32
G3. Splenic injury	5	5	5	5	5	5	5	5	5	5	5	5	5	5	5	5	5	5	5.00	0.00
G4. Liver injury	5	5	5	5	5	5	5	5	5	5	5	5	5	5	5	5	5	5	5.00	0.00
G5. Extrahepatic biliary lesion	5	5	5	5	5	5	5	5	5	5	5	5	5	5	5	5	5	5	5.00	0.00
G6. Pancreatic lesion	5	5	5	5	5	5	5	5	5	5	5	5	5	5	5	4	5	5	4.94	0.24
G7. Injury of hollow viscera	5	5	5	5	5	5	5	5	5	5	5	5	5	5	5	5	5	5	5.00	0.00
G8. Adrenal lesion	5	5	5	5	5	5	5	5	5	5	5	5	5	5	5	4	5	5	4.94	0.24
G9. Kidney injury	5	5	5	5	5	5	5	5	5	5	5	5	5	5	5	5	5	5	5.00	0.00
G10. Ureteral injury	5	5	5	5	5	5	5	5	5	5	5	5	5	5	5	5	5	5	5.00	0.00
G11. Bladder injury	5	5	5	5	5	5	5	5	5	5	5	5	5	5	5	5	5	5	5.00	0.00
G12. Urethral injury	5	5	5	5	5	4	5	5	5	5	5	5	5	5	5	4	5	5	4.89	0.32
G13. Uterine lesion (non-pregnant patient)	5	5	5	5	5	4	5	5	5	5	5	5	5	5	5	5	5	5	4.94	0.24
G14. Uterine lesion (pregnant patient)	5	5	5	5	5	4	5	5	5	5	5	5	5	5	5	5	5	5	4.94	0.24
H1. Cervical spine	5	5	5	5	5	5	5	5	5	5	5	5	5	5	5	5	5	5	5.00	0.00
H2. Dorso-lumbar spine	5	5	5	5	5	5	5	5	5	5	5	5	5	5	5	5	5	5	5.00	0.00
H3. Sacro-coccyx	5	5	5	5	5	5	5	5	5	5	5	5	5	5	5	5	5	5	5.00	0.00
H4. Pelvic girdle	5	5	5	5	5	5	5	5	5	5	5	5	5	5	5	5	5	5	5.00	0.00
I1. Upper limb fracture	5	5	5	5	5	5	5	5	5	5	5	5	5	5	5	5	5	5	5.00	0.00
I2. Fracture of the lower limb	5	5	5	5	5	5	5	5	5	5	5	5	5	5	5	5	5	5	5.00	0.00
I3. Peripheral vascular injury	5	5	5	5	5	5	5	5	5	5	5	5	5	5	4	5	5	5	4.94	0.24
Sum of scores	210	207	210	209	210	200	210	210	209	210	210	210	210	210	206	201	210	210	208.44	3.11

In both Delphi rounds, all sections received more than a good rating. In the first round, the overall mean score of the experts (n = 18) and the sum of scores for SR were 4.77 (range 1–5) and 257.56 (range 206–270), respectively (Table 1). In the second round, the overall mean score of the experts (*n* = 18) and the sum of scores for SR were 4.96 (range 4–5) and 208.44 (range 200–210), respectively (Table 2).

The experts’ overall mean score in the second Delphi round was higher than the overall mean score of the first round, along with a lower standard deviation value (19.71 in the first round versus 3.11 in the second round), highlighting the higher expert agreement on SR reached in the second round.

C*α* was 0.87 in the first Delphi round and increased to 0.97 in the second round.

## Discussion

To the best of our knowledge, this is the first comprehensive SR template proposed for the evaluation of polytrauma patients based on whole-body CT imaging. This template is currently the only one developed following the latest version of the AAST injury scoring scales [47] and the AO Spine Thoracolumbar and Subaxial Injury Classification systems [48]. The idea of using standardized scores (as per AAST and AO classifications) allows not only to use a common and easily understandable lexicon in the multidisciplinary team, but also to grade the level of injury and facilitate patient management.

Although the RSNA has proposed several SR templates for trauma, only the “CT Facial Bones—Trauma template [45] was reported on the RSNA website at the time of writing (7 January 2023) and is not based on internationally established classifications.

Our final template was divided into four sections, amounting to a total of as many as 118 items. Although this SR model may seem very long and prone to slowing down the reporting, it has been broadly demonstrated that SR can facilitate radiologist workflow by reducing reporting time [53]. Jorg et al. [53] found that SR for whole-body trauma CT examinations can add clinical value compared to FTR by reducing reporting time and increasing the level of detail for trauma CT studies. However, no data on how this model was built were provided [53].

Our template was established based on a multi-round Delphi method following in-depth discussion between expert radiologists in emergency imaging, leading to higher expert scores and internal consistency in the second Delphi round than in the first one. The panel experts showed a greater disagreement on the “Patient Clinical Data” and “Clinical Evaluation” sections, because of the opinion that in the emergency setting, time is critical and there may be no possibility to have all patient data available at the time of reporting [54–61]. However, after call conferences and once the option to fill in SR section fields partially had been clarified, all panelists expressed their agreement on this point.

The expert panel expressed a greater agreement on the “Imaging Protocol” and “Report” sections. Theoretically, the dissemination and sharing of the study protocol (including post-contrast phases) should ease the standardization and optimization of CT protocols in the emergency setting. Although each patient is different from another (especially in terms of hemodynamic stability and in relation to his/her anthropometric characteristics), the ability to act according to the rules of good conduct in the emergency setting could improve the quality of CT examinations and facilitate the interpretation of the results [62–74].

Regarding the “Report” section, the Delphi method has made it possible not only to collect key information based on established classifications, but also to address potentially critical issues. For instance, the information for the heart injury assessment (which, according to the AAST classification, also requires knowledge of data based on the electrocardiogram) may be unavailable at the time of the CT scan, so we decided to simplify the reporting of heart injury.

The chance of employing SR can assist the radiologist in the imaging assessment, allowing to detect imaging findings that might go unnoticed with FTR due to distraction [75–82]. On the other hand, a SR template allows a systematic search pattern that should avoid diagnostic errors. A retrospective analysis of 3,000 MRI studies using a checklist-template permitted to detect extraspinal findings in 28.5% of patients, which were not reported in the original FTR [75]. Similarly, the use of SR showed to increase the rate of identification of trauma-unrelated findings on cervical CT examinations [76]. Dendl et al. [77] showed that by using a SR template for major trauma CT examinations, radiology residents reached a higher diagnostic accuracy in the study setting than radiologists with 3 and 7 years of experience after board certification, highlighting the usefulness of SR as a guide for learning to correctly interpret and report imaging examinations.

The present SR has several strengths, as it is based on standardized terminology and structures, and according to Weiss et al. [78], it is a third-level SR. Furthermore, it has been validated by expert emergency radiologists by using a multi-round consensus-building Delphi exercise, and it has been built following the latest version of the AAST injury scoring scales and the AO Spine Thoracolumbar and Subaxial Injury Classification systems, with the goal to utilize a standardized language and improve communication within the trauma team.

This study has several limitations. Firstly, the same nationality of the panelists reduces the possibility of a wider view that could increase the consistency of the SR. Secondly, this SR template does not arise from a multidisciplinary evaluation, but only from a panel of radiologists. Thirdly, we did not test the clinical impact of this SR in a real-life emergency setting, and we did not assess the actual turnaround time of polytrauma CT examinations using this SR template, which is quite lengthy due to the complex nature of polytrauma CT reporting and could therefore be rather time-consuming. However, the highly modular and standardized structure of this SR from head to toe could be expected to keep reporting times within acceptable limits after a proper learning period, and in the extreme, it could lend itself as a conceptual framework for the development of more streamlined SR models based on the same rigorous semantics.

## Conclusion

We developed a comprehensive SR model for whole-body CT examinations of polytrauma patients, based on a multi-round Delphi consensus of emergency radiology experts and following current AAST and AO Spine criteria. It could be expected that this SR will set a standard for improving the quality of radiological reports and the communication between all physicians involved in the trauma team.

## Supplementary Information

Below is the link to the electronic supplementary material.

Supplementary file1 (DOCX 83 kb)

## Abbreviations

AAST: American Association for the Surgery of Trauma
ATLS: Advanced Trauma Life Support
BMI: Body Mass Index
BSA: Body Surface Area
C*α*: Cronbach's correlation coefficient alpha
ESER: European Society of Emergency Radiology
FTR: Free text report
GCS: Glasgow Coma Score
HIS: Hospital Information System
HTML5: HyperText Markup Language version 5
IHE: Integrating Healthcare Enterprise
MRRT: Management of Radiology Report Templates
RIS: Radiology Information System
RSNA: Radiological Society of North America
SIRM: Italian Society of Medical and Interventional Radiology
SR: Structured reporting
