# Supplementary material for: Structured reporting of computed tomography in the polytrauma patient assessment: a Delphi consensus proposal
Source: Radiol Med. 2023 Jan 19;128(2):222–33. doi: 10.1007/s11547-023-01596-8 (PMC9938818; doi:10.1007/s11547-023-01596-8)
Supplement: Supplementary file 1 — Supplementary file1 (DOCX 83 kb) [file 11547_2023_1596_MOESM1_ESM.docx]

**PATIENT CLINICAL DATA (automatically imported from HIS/RIS)**

| *FIELD* | *DETAIL* | *ADMITTED VALUES* |
| --- | --- | --- |
| ANTHROPOMETRIC DATA | | |
| Weight | *(if available)* | *(kg) [Numeric]* |
| Height | *(if available)* | *(cm) [Numeric]* |
| BMI |  | *[Numeric]* (automatically calculated) |
| BSA |  | *[Numeric]* (automatically calculated) |
| Age |  | (years) *[Numeric]* |
| Age class |  | - 0-49 years old - 50 – 69 years old - ≥70 years old |

**CLINICAL EVALUATION (automatically imported from HIS/RIS)**

| *FIELD* | *DETAIL* | *ADMITTED VALUES* |
| --- | --- | --- |
| CLINICAL INFORMATION | | |
| Type of trauma | Known | *[Yes/No]* |
|  | Road accident   - High speed impact (>85km/h) or vehicle deformation >50cm or passenger compartment intrusion >30cm | *[Yes/No]* |
|  |  |  |
|  | - People deceased in the same vehicle or ejection outside the vehicle | *[Yes/No]* |
|  | - Pedestrian hit and thrown by a car or rolling | *[Yes/No]* |
|  | - Fall from motorbike with motorbike / driver separation at speed >30km/h | *[Yes/No]* |
|  | - Crushing | *[Yes/No]* |
|  | - Penetrating trauma | *[Yes/No]* |
|  | - Precipitation   (if Yes, fall from height >5m [Yes/No]) | *[Yes/No]* |
|  | - Burn   (if Yes, burn >30% of body surface *[*Yes/No*]*) | *[Yes/No]* |
| Pathophysiological criteria | Intubated | *[Yes/No]* |
|  | GCS <13 | *[Yes/No]* |
|  | Systolic arterial pressure <90 mmHg | *[Yes/No]* |
|  | Respiratory rate >29/min | *[Yes/No]* |
|  | Respiratory rate <10/min | *[Yes/No]* |
|  | Oxygen saturation (in room air) <90% | *[Yes/No]* |
|  | Sites involved | - Head *[Yes/No]* - Neck *[Yes/No]* - Chest *[Yes/No]* - Abdomen *[Yes/No]* - Spine *[Yes/No]* - Limbs *[Yes/No]* |
| Anatomical criteria (based on clinical examination) | Side deficit | Yes/No |
|  | Sensory/motor deficit | Yes/No |
|  | Chest trauma with mobile parietal flap | Yes/No |
|  | Penetrating injury | Yes/No |
|  | Proximal fracture of 2 or more long bones | Yes/No |
|  | Amputation proximal to elbow or knee | Yes/No |
| Notes | *[Free text]* | |

**IMAGING TECHNIQUE**

| *FIELD* | *DETAIL* | *ADMITTED VALUES* |
| --- | --- | --- |
| IMAGING DATA | | |
| Examination date | Date | Date *[dd/mm/yyyy]* |
| Clinical indication | Polytrauma | |
| Scanner brand and model |  | *[Free text]* |
| Scan protocol | Number of detector rows | *[Numeric]* |
|  | Precontrast scan  ***(details visible only if “Yes”)*** | Dual energy *[Yes/No]*  Slice thickness (mm) *[Numeric]*  Convolution kernel(s) *[Free text]*  Body area *[Multiple choice]*:   - head - neck - chest - abdomen - upper limbs - lower limbs |
|  | Post-contrast scan  ***(details repeatable for each post-contrast scan)*** | Number of scans *[Numeric]*  Type (early arterial, late arterial, venous, late, urographic, other *[specify: Free text]*)  Dual energy *[Yes/No]*  Slice thickness (mm) *[Numeric]*  Convolution kernel(s) *[Free text]*  Body area *[Multiple choice]*:   - head - neck - chest - abdomen - upper limbs - lower limbs |
| Radiation exposure | Class of radiation exposure | *[Numeric]* |
| Notes | *[Free text]* | |
| CONTRAST MEDIUM | | |
| Use of contrast medium  (visible only if “Yes”) | Yes/No | |
|  | Active principle | - Iobitridol - Iodixanol - Iohexol - Iomeprol - Iopromide - Ioversol |
|  | Commercial name | *[Free text]* |
|  | Volume | *[Numeric]* (ml) |
|  | Flow rate | *[Numeric]* (ml/sec) |
|  | Concentration | *[Numeric]* (mg I/ml) |
|  | Notes | *[Free text]* |
| ADVERSE EVENTS | | |
| ONGOING adverse events  (visible only if “Yes”) | Yes/No | |
|  | Date and hour of event | *[dd/mm/yyyy, hour]* |
|  | Degree | - Mild - Moderate - Severe |
|  | Time of onset | - Early - Late   Minutes *[Numeric]* (optional) |
|  | Type | ALLERGIC / ALLERGIC-LIKE  **Mild**   - Sparse wheals/itch - Skin edema - Mild itching / feeling like ”velvet in the throat“ - Nasal congestion - Sneezing - Conjunctivitis - Rhinorrhea   **Moderate**   - Diffuse wheals/intense itch - Diffuse skin edema - Facial edema without dyspnea - Feeling of choking or hoarseness - Wheezing / mild bronchospasm without hypoxia   **Severe**   - Dyspnea - Erythema – diffuse mucocutaneous symptoms - Laryngeal edema with stridor and/or hypoxia - Wheezing / bronchospasm - Significant hypoxia - Anaphylactic shock (severe hypotension and brady-tachyarrhythmia)   NON-ALLERGIC  **Mild**   - Mild nausea/limited vomiting - Transient chills / heat / redness - Headache / dizziness / anxiety / altered taste - Slight increase in blood pressure - Self-limiting vasovagal reaction   **Moderate**   - Prolonged nausea/vomiting - Elevated arterial blood pressure - Isolated chest pain - Vasovagal reaction   **Severe**   - Treatment-refractory vasovagal reaction - Arrhythmia - Convulsions - Severe arterial hypertension   CONTRAST MEDIUM EXTRAVASATION |
|  | Type of treatment | - Wait and see - Drug therapy (specify in “Notes” field) - Anesthesiologist’s intervention required |
|  | Event resolution | - Spontaneous - After treatment - After hospitalization - Other *[Free text]* |
| Notes | *[Free text]* | |

**REPORT**

| *FIELD* | *DETAIL* | *ADMITTED VALUES* |
| --- | --- | --- |
| 1. HEAD | | |
| SKULL | | |
| Fracture  *(details visible only if “Yes”)* | Yes/No | |
|  | Site | - Frontal - Parietal - Temporal - Occipital |
|  | Fragment dislocation | Yes/No, specify *[Free text]* |
| Extra-axial compartment | Unremarkable | Yes/No |
| Subdural hematoma  *(details visible only if “Yes”)* | Yes/No | |
|  | Site | - Frontal - Parietal - Temporal - Occipital - Subtentorial |
|  | Side | - Right - Left - Bilateral |
|  | Thickness | mm *[Numeric]* |
| Epidural hematoma  *(details visible only if “Yes”)* | Yes/No | |
|  | Site | - Frontal - Parietal - Temporal - Occipital - Subtentorial - Vertex |
|  | Side | - Right - Left - Bilateral |
|  | Thickness | mm *[Numeric]* |
| Subarachnoid hemorrhage  *(details visible only if “Yes”)* | Yes/No | |
|  | Site | - Focal - Diffuse - Supratentorial - Subtentorial |
| BRAIN TISSUE  *(details visible only if “Yes”)* | Unremarkable | Yes/No |
|  | Site | - Supratentorial - Subtentorial |
| Focal lesions  *(details visible only if “Yes”)* | Yes/No | |
|  | Location of largest lesion | - Frontal (right/left) - Parietal (right/left) - Temporal (right/left) - Occipital (right/left) - Cerebellar (right/left) |
|  | Type | - Edema - Edema and blood |
| Diffuse lesions | Yes/No | |
|  | Type | - Diffuse axonal damage (Yes/No) - Other *[Free text]* |
| MIDLINE |  | |
| Midline shift  *(details visible only if “Yes”)* | Yes/No | |
|  | Side | - Right - Left |
|  | Size | mm *[Numeric]* |
| Herniation  *(details visible only if “Yes”)* | Yes/No | |
|  | If Yes, specify type: | - Subfalcine - Transtentorial, temporal or uncal - Central or descending transtentorial - Ascending transtentorial - Tonsillar - Extracranial (transcalvarium) |
| VENTRICULAR SYSTEM |  | |
| Supratentorial | Size | - Normal - Increased - Decreased |
|  | Intraventricular hemorrhage | Yes/No |
| Subtentorial | Size | - Normal - Increased - Decreased |
|  | Intraventricular hemorrhage | Yes/No |
| NOTES | *[Free text]* | |

| ASSOCIATED FINDINGS | | |
| --- | --- | --- |
| Maxillo-facial trauma  *details visible only if “Yes”)* | Yes/No  (specify) | *[Free text]* |
| Craniocervical junction trauma  *details visible only if “Yes”)* | Yes/No  (specify) | *[Free text]* |
| Incidental findings | Yes/No  (specify) | *[Free text]* |
| NOTES | *[Free text]* | |

| *FIELD* | *DETAIL* | *ADMITTED VALUES* |
| --- | --- | --- |
| 1. NECK   **AAST injury severity scoring 2021 -* [*https://www.aast.org/resources-detail/injury-scoring-scale*](https://www.aast.org/resources-detail/injury-scoring-scale) | | |
| Cervical vascular organ injury  **Note: Increase one grade for multiple grade III or IV injuries involving more than 50% vessel circumference. Decrease one grade for less than 25% vessel circumference disruption for grade IV or V* | Yes/No | |
|  | Description of injury | - Dissection (Yes/No) - Thrombosis (Yes/No) - Pseudoaneurysm (Yes/No) - Active bleeding (Yes/No) |
|  |  | Grade |
|  | - Thyroid vein - Common facial vein - External jugular vein - Non-named arterial/venous branches | I |
|  | - External carotid arterial branches (ascending pharyngeal, superior thyroid, Iingual, facial maxillary, occipital, posterior auricular) - Thyrocervical trunk or primary branches - Internal jugular vein - External carotid artery | II |
|  | - Subclavian vein - Vertebral artery - Common carotid artery | III |
|  | - Subclavian artery | IV |
|  | - Internal carotid artery (extracranial) | V |
| Notes | *[Free text]* | |

| *FIELD* | *DETAIL* | *ADMITTED VALUES* |
| --- | --- | --- |
| 1. CHEST | | |
| Pleural effusion | Yes/No | If Yes, specify:   - site (right, left, bilateral) - hemothorax *[Yes/No]* |
|  | Amount | *[Free text]* |
| Pneumothorax | Yes/No | If Yes, specify:   - site (right, left, bilateral) - hypertensive pneumothorax *[Yes/No]* |
|  | Amount | *[Free text]* |
| Pneumomediastinum | Yes/No | If Yes, specify *[Free text]* |
|  | Amount | *[Free text]* |
| Pericardial effusion | Yes/No | If Yes, specify:   - site *[Free text]* - hemopericardium *[Yes/No]* - cardiac tamponade *[Yes/No]* |
|  | Amount | *[Free text]* |
| Tracheobronchial lesions | Yes/No | If Yes, specify:   - site *[Free text]* - penetrating / iatrogenic / blunt trauma - laceration / parietal tear |
| Notes | *[Free text]* | |
| ORGAN-SPECIFIC INJURIES  **AAST injury severity scoring 2021 -* [*https://www.aast.org/resources-detail/injury-scoring-scale*](https://www.aast.org/resources-detail/injury-scoring-scale) | | |
| Chest wall injury  **Note: This scale is confined to the chest wall alone and does not reflect associated internal or abdominal injuries* | Yes/No | |
|  | Injury Type | Description of Injury / Grade |
|  | - Contusion | Any size / I |
|  | - Laceration | - Skin & subcutaneous / I - Skin, subcutaneous and muscle / II - Full thickness including pleural penetration / III - Avulsion of chest wall tissues with underlying rib fractures, unilateral flail chest (≥3 ribs) / IV |
|  | - Fracture | - < 3 ribs closed / I - nondisplaced clavicle closed / I - ≥3 adjacent ribs, closed / II - Open or displaced clavicle / II - Non-displaced sternum, closed / II - Scapular body, open or closed / II - Open or displaced sternum / III - Flail sternum / III - Unilateral flail segment (<3 ribs) / III - Bilateral flail chest (≥3 ribs on both sides) / IV - V (fracture) |
|  | Notes | *[Free text]* |
| Heart injury | Yes/No | |
|  | Description of injury | *[Free text]* |
| Lung injury | Yes/No | |
|  | Injury Type | Description of Injury / Grade |
|  | - Contusion | - Unilateral, <1 lobe / I - Unilateral, single lobe / II - Unilateral, >1 lobe / III |
|  | - Laceration | - Simple pneumothorax / II - Persistent (>72hrs) air leak from distal airway / III - Major (segmental or lobar) air leak / IV |
|  | - Hematoma | - Nonexpanding intraparenchymal / III - Expanding intraparenchymal / IV |
|  | - Vascular | - Primary branch intrapulmonary vessel disruption / IV - Hilar vessel disruption / V - Total uncontained transection of pulmonary hilum / VI |
|  | Notes | *[Free text]* |
| Thoracic Vascular Injury  **Note: Increase one grade for multiple grade III or IV injuries if more than 50% circumference; decrease one grade for grade IV injuries if less than 25% circumference* | Yes/No | |
|  | Description of injury | - Dissection *[Yes/No]* - Thrombosis *[Yes/No]* - Pseudoaneurysm *[Yes/No]* - Active bleeding *[Yes/No]* |
|  |  | Grade |
|  | - Intercostal artery/vein - Internal mammary artery/vein - Bronchial artery/vein - Esophageal artery/vein - Hemiazygos vein - Unnamed artery/vein | I |
|  | - Azygos vein - Internal jugular vein - Subclavian vein - Innominate vein | II |
|  | - Carotid artery - Innominate artery - Subclavian artery | III |
|  | - Thoracic aorta, descending - Inferior vena cava (intrathoracic) - Pulmonary artery, primary intraparenchymal branch - Pulmonary vein, primary intraparenchymal branch | IV |
|  | - Thoracic aorta, ascending and arch - Superior vena cava - Pulmonary artery, main trunk - Pulmonary vein, main trunk | V |
|  | - Uncontained total transection of thoracic aorta or pulmonary hilum | VI |
|  | Notes | *[Free text]* |
| Esophagus injury | Yes/No | |
|  | Injury type | - Contusion / Hematoma *[Yes/No]* - Laceration *[Yes/No]* - Segmental loss or devascularization *[Yes/No]* |
|  | Notes | *[Free text]* |
| Diaphragm injury | Yes/No | |
|  | Side | Right / Left |
|  | Injury type | - Contusion *[Yes/No]* - Hematoma *[Yes/No]*   if Yes: active bleeding *[Yes/No]*   - Laceration *[Yes/No]* - Visceral herniation *[Yes/No]*   if Yes: vascular complications *[Yes/No]* |
|  | Notes | *[Free text]* |
| Notes | *[Free text]* | |

| *FIELD* | *DETAIL* | *ADMITTED VALUES* |
| --- | --- | --- |
| 1. ABDOMEN | | |
| Peritoneal effusion | Yes/No | If Yes, specify:   - Site   -perihepatic  -perisplenic  -right paracolic gutter  -left paracolic gutter  -pelvic   - Hemoperitoneum *[Yes/No]* |
|  | Amount | *[Free text]* |
| Pneumoperitoneum | Yes/No | If Yes, specify site *[Free text]* |
|  | Amount | *[Free text]* |
| ORGAN-SPECIFIC INJURIES  **AAST injury severity scoring 2021 -* [*https://www.aast.org/resources-detail/injury-scoring-scale*](https://www.aast.org/resources-detail/injury-scoring-scale) | | |
| Abdominal vascular injury  **Note: This classification system is applicable to extraparenchymal vascular injuries. If the vessel injury is within 2 cm of the organ parenchyma, refer to specific organ injury scale. Increase one grade for multiple grade III or IV injuries involving >50% vessel circumference. Downgrade one grade if <25% vessel circumference laceration for grades IV or V.* | Yes/No | |
|  | Description of injury | - Dissection *[Yes/No]* - Thrombosis *[Yes/No]* - Pseudoaneurysm *[Yes/No]* - Active bleeding *[Yes/No]* |
|  |  | Grade |
|  | - Non-named superior mesenteric artery or superior mesenteric vein branches - Non-named inferior mesenteric artery or inferior mesenteric vein branches - Phrenic artery or vein - Lumbar artery or vein - Gonadal artery or vein - Ovarian artery or vein - Other non-named small arterial or venous structures requiring ligation | I |
|  | - Right, left, or common hepatic artery - Splenic artery or vein - Right or left gastric arteries - Gastroduodenal artery - Inferior mesenteric artery, or inferior mesenteric vein, trunk - Primary named branches of mesenteric artery (e.g., ileocolic artery) or mesenteric vein - Other names abdominal vessels requiring ligation or repair | II |
|  | - Superior mesenteric vein, trunk - Renal artery or vein - Iliac artery or vein - Hypogastric artery or vein - Vena cava, infrarenal | III |
|  | - Superior mesenteric artery, trunk - Celiac axis proper - Vena cava, suprarenal and infrahepatic - Aorta (infrarenal) | IV |
|  | - Portal vein - Extraparenchymal hepatic vein - Vena cava, retrohepatic or suprahepatic - Aorta (suprarenal, subdiaphragmatic) | V |
|  | Notes | *[Free text]* |
| Spleen injury  **Note: Vascular injury is defined as a pseudoaneurysm or arteriovenous fistula and appears as a focal collection of vascular contrast that decreases in attenuation with delayed imaging. Active bleeding from a vascular injury presents as vascular contrast, focal or diffuse, that increases in size or attenuation in delayed phase. Vascular thrombosis can lead to organ infarction. More than one grade of splenic injury may be present and should be classified by the higher grade of injury. Advance one grade for multiple injuries up to grade III.* | Yes/No | |
|  | Description of injury | Grade |
|  | - Hematoma | - Subcapsular, <10% surface area / I - Subcapsular, 10%-50% surface area / II intraparenchymal, <5cm in diameter / II - Subcapsular, >50% surface area / III, ruptured subcapsular or intraparenchymal, ≥ 5cm / III |
|  | - Laceration | - Capsular tear, <1cm parenchymal depth / I - 1-3cm parenchymal depth / II - >3 cm parenchymal depth / III - Laceration involving segmental or hilar vessels producing major devascularization (>25% of spleen) / IV - Completely shattered spleen / V |
|  | - Vascular | - Any injury in the presence of a splenic vascular injury or active bleeding confined with splenic capsule / IV - Any injury in the presence of a splenic vascular injury with active bleeding extended beyond the spleen into the peritoneum / V |
|  | Notes | *[Free text]* |
| Liver injury  **Note: Vascular injury is defined as a pseudoaneurysm or arteriovenous fistula and appears as a focal collection of vascular contrast that decreases in attenuation with delayed imaging. Active bleeding from a vascular injury presents as vascular contrast, focal or diffuse, that increases in size or attenuation in delayed phase. Vascular thrombosis can lead to organ infarction. More than one grade of liver injury may be present and should be classified by the higher grade of injury. Advance one grade for multiple injuries up to grade III.* | Yes/No | |
|  | Description of injury | Grade |
|  | - Hematoma | - Subcapsular, <10% surface area / I - Subcapsular, 10% to 50% surface area, intraparenchymal <10cm in diameter / II - Subcapsular, >50% surface area of ruptured subcapsular or parenchymal hematoma / III |
|  | - Laceration | - <1cm parenchymal depth / I - 1-3 parenchymal depth and ≤ 10cm in length / II - >3cm parenchymal depth / III - Parenchymal disruption involving 25% to 75% hepatic lobe / IV - Parenchymal disruption involving >75% of hepatic lobe / V |
|  | - Vascular | - Any injury in the presence of a liver vascular injury or active bleeding contained within liver parenchyma / III - Active bleeding extending beyond the liver parenchyma into the peritoneum / IV - Juxtahepatic venous injuries to include retrohepatic vena cava and central major hepatic veins / V |
|  | Notes | *[Free text]* |
| Extrahepatic biliary tree injury | Yes/No | |
|  | Description of injury | *[Free text]* |
| Pancreas Injury | Yes/No | |
|  | Description of injury | - Contusion *[Yes/No]* - Hematoma *[Yes/No]* - If Yes: active bleeding *[Yes/No]* - Laceration *[Yes/No]* - Fracture *[Yes/No]* |
|  | Notes | *[Free text]* |
| Hollow viscus injury | Yes/No | |
|  | Site | - Stomach - Duodenum - Small bowel - Large bowel - Rectum |
|  | Description of injury | - Contusion / Hematoma *[Yes/No]* - Partial thickness laceration *[Yes/No]* - Rupture / segmental loss *[Yes/No]* |
|  | Notes | *[Free text]* |
| Adrenal organ injury  **Note: Advance one grade for bilateral lesions up to grade V* | Yes/No | |
|  | Description of injury | Grade |
|  | - Contusion | I |
|  | - Laceration | II/III |
|  | - >50% parenchymal destruction | IV |
|  | - Total parenchymal destruction (including massive intraparenchymal hemorrhage) - Avulsion from blood supply | V |
|  | Notes | *[Free text]* |
| Kidney injury  **Note: Vascular injury is defined as a pseudoaneurysm or arteriovenous fistula and appears as a focal collection of vascular contrast that decreases in attenuation with delayed imaging. Active bleeding from a vascular injury presents as vascular contrast, focal or diffuse, that increases in size or attenuation in delayed phase. Vascular thrombosis can lead to organ infarction. More than one grade of kidney injury may be present and should be classified by the higher grade of injury. Advance one grade for multiple injuries up to grade III.* | Yes/No | |
|  | Description of injury | Grade |
|  | - Contusion | - Parenchymal without laceration / I |
|  | - Hematoma | - Subcapsular / I - Perirenal hematoma confined to Gerota fascia / II |
|  | - Laceration | - ≤1.0cm parenchymal depth of renal cortex without urinary extravasation / II - >1.0cm parenchymal depth of renal cortex without collecting system rupture or urinary extravasation / III - Parenchymal laceration extending through renal cortex, medulla, and collecting system with urinary extravasation / IV - Renal pelvis laceration and/or complete ureteropelvic disruption / IV - Completely shattered kidney / V |
|  | - Vascular | - Any injury in the presence of a kidney vascular injury or active bleeding contained within Gerota fascia / III - Segmental renal artery or vein injury / IV - Active bleeding beyond Gerota fascia into retroperitoneum or peritoneum / IV - Segmental or complete kidney infarction(s) due to vessel thrombosis without active bleeding / IV - Main renal artery or vein laceration or avulsion of hilum / V - Devascularized kidney with active bleeding / V |
|  | Notes | *[Free text]* |
| Ureter injury  **Note: Advance one grade for bilateral injuries up to grade III* | Yes/No | |
|  | Description of injury |  |
|  | - Hematoma | - Contusion or hematoma |
|  | - Laceration | - Incomplete transection - Complete transection |
|  | Notes | *[Free text]* |
| Bladder injury  **Note: Advance one grade for multiple lesions up to grade III* | Yes/No | |
|  | Description of injury | Grade |
|  | - Hematoma | - Contusion, intramural hematoma |
|  | - Laceration | - Extraperitoneal bladder wall laceration <2cm / II - Extraperitoneal (≥2cm) or intraperitoneal (<2cm) bladder wall laceration / III - Intraperitoneal bladder wall laceration ≥2cm / IV - Intraperitoneal or extraperitoneal bladder wall laceration extending into the bladder neck or ureteral orifice (trigone) / V |
|  | Notes | *[Free text]* |
| Urethra injury | Yes/No | |
|  | Description of injury | Grade |
|  | - Partial disruption | - Extravasation of urethrography contrast at injury site with visualization in the bladder / III |
|  | - Complete disruption | - Extravasation of urethrography contrast at injury site without visualization in the bladder; <2cm of urethra separation / IV - Complete transection with ≥2cm urethral separation, or extension into the prostate or vagina / V |
|  | Notes | *[Free text]* |
| Uterus (nonpregnant) injury  **Note: Advance one grade for multiple injuries up to grade III* | Yes/No | |
|  | Description of injury | Grade |
|  | - Contusion / Hematoma | I |
|  | - Superficial laceration (<1cm) | II |
|  | - Deep laceration (≥1cm) | III |
|  | - Laceration involving uterine artery | IV |
|  | - Avulsion/devascularization | V |
|  | Notes | *[Free text]* |
| Uterus (pregnant) injury  **Note: Advance one grade for multiple injuries up to grade III* | Yes/No | |
|  | Description of injury | Grade |
|  | - Contusion or hematoma (without placental abruption) | I |
|  | - Superficial laceration (<1cm) or partial placental abruption <25% - Deep laceration (≥1cm) occurring in second trimester or placental abruption >25% but <50% | II |
|  | - Deep laceration (≥1cm) in third trimester - Laceration involving uterine artery | III |
|  | - Deep laceration (≥1cm) with >50% placental abruption | IV |
|  | - Uterine rupture in second or third trimester - Complete placental abruption | V |
|  | Notes | *[Free text]* |
| Notes | *[Free text]* | |

| *FIELD* | *DETAIL* | *ADMITTED VALUES* |
| --- | --- | --- |
| 1. SPINE - AO Spine classification https://aospine.aofoundation.org/clinical-library-and-tools/ao-spine-classification-systems | | |
| CERVICAL SPINE | Yes/No | |
| Upper cervical injury | Yes/No |  |
| I: Occipital Condyle and Craniocervical junction *(details visible only if “Yes”)*    II: C1 Ring and C1-2 Joint  *(details visible only if “Yes”)*  III: C2 and C2-3 Joint  *(details visible only if “Yes”)* | Yes/No |  |
|  | Isolated bony injury (condyle) | Type A |
|  | - Nondisplaced ligamentous injury (craniocervical) | Type B |
|  | Any injury with displacement on spinal imaging | Type C |
|  | Yes/No |  |
|  | Isolated bony only (arch) | Type A |
|  | Ligamentous injury (transverse atlantal ligament) | Type B |
|  | Atlantoaxial instability / Translation in any plain | Type C |
|  | Yes/No |  |
|  | Bony injury only (without ligamentous, tension band, discal injury) | Type A |
|  | Tension band / Ligamentous injury with or without bony injury | Type B |
|  | Any injury that leads to vertebral body translation in any directional plane | Type C |
| Subaxial injury  *(details visible only if “Yes”)*  Type A: Compression Injuries  *(details visible only if “Yes”)*  **Note:* compression of the anterior structures or fractures of the spinal process that are mechanically insignificant  Type B: Tension Band Injuries  *(details visible only if “Yes”)*  **Note:* affect the tension bands anterior or posterior to the cervical spine. Any type B injuries that also have translation are automatically type C injuries | Yes/No | |
|  | Location | - C3 - C4 - C5 - C6 - C7 |
|  | Yes/No |  |
|  | No injury or minor injury (lamina or spinous process fracture) | A0 |
|  | Wedge compression (involves one endplate but not the posterior wall) | A1 |
|  | Split (involves both endplates but not the posterior wall) | A2 |
|  | Incomplete burst (involves one endplate and the posterior wall) | A3 |
|  | Complete burst (involves both endplates and the posterior wall) | A4 |
|  | Yes/No |  |
|  | Posterior tension band injury (bony) with physical separation between fractured bony structures (anterior structures may also be included) | B1 |
|  | Posterior tension band injury (bony, capsuloligamentous, ligamentous) with complete disruption of the capsuloligamentous or bony capsuloligamentous structures of the posterior aspect (anterior structures may also be included) | B2 |
|  | Anterior tension band injury with physical separation between anterior structures and tethering of the posterior structures | B3 |
| Type C: Translation Injuries  **Note: in any direction* | Yes/No |  |
| Type F: Facet Injuries  *(details visible only if “Yes”)* | Yes/No |  |
|  | Nondisplaced facet fracture | F1 |
|  | Facet fracture with potential for instability | F2 |
|  | Floating lateral mass (due to disruption of pedicle and lamina) | F3 |
|  | Pathologic subluxation or perched/dislocated facet | F4 |
| Bilateral Injuries | Yes/No | BL |
| Notes | *[Free text]* | |
| DORSO-LUMBAR SPINE  Type A: Compression Injuries  *(details visible only if “Yes”)* | Yes/No | |
|  | Location | - T1 - T2 - T3 - T4 - T5 - T6 - T7 - T8 - T9 - T10 - T11 - T12 |
|  | Yes/No | |
|  | Minor nonstructural fracture (transverse or spinous process) | A0 |
|  | Wedge compression (involves one endplate but not the posterior wall) | A1 |
|  | Split (involves both endplates but not the posterior wall) | A2 |
|  | Incomplete burst (involves one endplate and the posterior wall) | A3 |
|  | Complete burst (involves both endplates and the posterior wall) | A4 |
| Type B: Distraction Injuries  *(details visible only if “Yes”)* | Yes/No |  |
|  | Transosseous posterior tension band disruption - Chance fracture | B1 |
|  | Posterior tension band disruption (bony and/or ligamentary, together with a Type A fracture. Type A fracture should be classified separately) | B2 |
|  | Hyperextension (injury through the disc or vertebral body) | B3 |
| Type C: Translation Injuries  **Note:* Displacement/ Dislocation (there are no subtypes because various configurations are possible due to dissociation/ dislocation. Can be combined with subtypes of A or B) | Yes/No | |
| Notes | *[Free text]* | |
| SACRUM AND COCCYX | Yes/No | |
| Type A: lower sacrococcygeal fractures  *(details visible only if “Yes”)* | Yes/No |  |
|  | Coccygeal or compression vs ligamentous avulsion fractures | A1 |
|  | Non-displaced transverse fractures below the S-I joint | A2 |
|  | Displaced transverse fractures below the S-I joint | A3 |
| Type B: Posterior Pelvic Injuries  *(details visible only if “Yes”)* | Yes/No |  |
|  | Central Fracture— involves spinal canal | B1 |
|  | Transalar Fracture— does not involve foramina or spinal canal | B2 |
|  | Transforaminal Fracture—involves foramina but not spinal canal | B3 |
| Type C: Spino-Pelvic Injuries *(details visible only if “Yes”)* | Yes/No |  |
|  | Nondisplaced sacral U-type variant | C0 |
|  | Sacral U-type variant without posterior pelvic instability | C1 |
|  | Bilateral complete Type B injuries without transverse fracture | C2 |
|  | Displaced U-type sacral fracture | C3 |
| Notes | *[Free text]* | |
| 61 PELVIC GIRDLE  AO/OTA Fracture Classification  <https://classification.aoeducation.org> | Yes/No | |
|  | **61 A: stable** (intact posterior arch) | - 61A1: avulsion injury - 61A2: iliac-wing or anterior-arch fracture due to a direct blow - 61A3: transverse sacrococcygeal fracture |
|  | **61 B: partially stable** (incomplete disruption of the posterior arch) | - 61B1: open-book injury (external rotation) - 61B2: lateral-compression injury (internal rotation) - 61B3: bilateral |
|  | **61 C: unstable** (complete disruption of the posterior arch) | - 61C1: unilateral - 61C2: bilateral, with one side type B, one side type C - 61C3: bilateral |
| Notes | *[Free text]* | |

| *FIELD* | *DETAIL* | *ADMITTED VALUES* |
| --- | --- | --- |
| 1. LIMBS AO/OTA Fracture Classification   <https://classification.aoeducation.org> | | |
| UPPER LIMB FRACTURE  *(details visible only if “Yes”)* | Yes/No | |
| Location Bone involved | **Bone portion** | **Group** |
| 1 Humerus | **11 Proximal end segment** | **11A Extraarticular, unifocal, 2-part**   - 11A1 Tuberosity - 11A2 Surgical neck - 11A3 Vertical   **11B Extraarticular, bifocal, 3-part**   - 11B1 Surgical neck   **11C Articular or 4-part**   - 11C1 Anatomical neck - 11C3 Anatomical neck associated with metaphyseal fracture |
|  | **12 Diaphyseal segment** | **12A Simple**   - 12A1* Spiral - 12A2* Oblique (≥ 30°) - 12A3* Transverse (< 30°)   **12B Wedge**   - 12B2* Intact wedge - 12B3* Fragmentary wedge   **12C Multifragmentary**   - 12C2* Intact segmental - 12C3* Fragmentary segmental   *** Qualifications**: *12A and 12B: a Proximal 1/3, b Middle 1/3, c Distal 1/3 12C: i Proximal diaphyseal-metaphyseal, j Pure diaphyseal, k Distal diaphyseal-metaphyseal* |
|  | **13 Distal end segment** | **13A Extraarticular**   - 13A1 Avulsion - 13A2 Simple - 13A3 Wedge or multifragmentary   **13B Partial articular**   - 13B1 Lateral sagittal - 13B2 Medial sagittal - 13B3 Frontal (coronal) plane   **13C Complete articular**   - 13C1 Simple articular, simple metaphyseal - 13C2 Simple articular, wedge or multifragmentary metaphyseal - 13C3 Multifragmentary articular, wedge or multifragmentary metaphyseal |
| 2R Radius | **2R1 Proximal end segment** | **2R1A Extraarticular**   - 2R1A1 Avulsion of bicipital tuberosity - 2R1A2 Neck, simple - 2R1A3 Neck, multifragmentary   **2R1B Partial articular**   - 2R1B1 Simple - 2R1B3 Fragmentary   **2R1C Complete articular**   - 2R1C1 Simple - 2R1C3 Multifragmentary |
|  | **2R2 Diaphyseal segment** | **2R2A Simple**   - 2R2A1* Spiral - 2R2A2* Oblique (≥30°) - 2R2A3* Transverse (<30°)   **2R2B Wedge**   - 2R2B2* Intact wedge - 2R2B3* Fragmentary wedge   **2R2C Multifragmentary**   - 2R2C2* Intact segmental - 2R2C3* Fragmentary segmental   *** Qualifications**: *2R2A and 2R2B: a Proximal 1/3, b Middle 1/3, c Distal 1/3 2R2C: i Proximal diaphyseal-metaphyseal, j Pure diaphyseal, k Distal diaphyseal-metaphyseal* |
|  | **2R3 Distal end segment** | **2R3A Extraarticular**   - 2R3A1 Radial styloid avulsion - 2R3A2 Simple - 2R3A3 Wedge or multifragmentary   **2R3B Partial articular**   - 2R3B1 Sagittal - 2R3B2 Dorsal rim (Barton's) - 2R3B3 Volar rim (reverse Barton's, Goyrand-Smith's II)   **2R3C Complete articular**   - 2R3C1 Simple articular and metaphyseal - 2R3C2 Multifragmentary metaphyseal - 2R3C3 Multifragmentary articular, simple or multifragmentary metaphyseal |
| 2U Ulna | **2U1 Proximal end segment** | **2U1A Extraarticular**   - 2U1A1 Avulsion of triceps insertion - 2U1A2 Simple metaphyseal - 2U1A3 Multifragmentary metaphyseal   **2U1B Partial articular**   - 2U1B1* Olecranon - 2U1B2* Coronoid   **2U1C Complete articular**   - 2U1C3* Olecranon and coronoid   ***Qualifications:** *B1: d Simple, e Multifragmentary B2: n Involving sublime facet, o Tip (avulsion), p <50%, q ≥50% C3: d Simple, r Multifragmentary olecranon, s Multifragmentary involving coronoid process* |
|  | **2U2 Diaphyseal segment** | **2U2A Simple**   - 2U2A1* Spiral - 2U2A2* Oblique (≥30°) - 2U2A3* Transverse (<30°)   **2U2B Wedge**   - 2U2B2* Intact wedge - 2U2B3* Fragmentary wedge   **2U2C Multifragmentary**   - 2U2C2* Intact segmental - 2U2C3* Fragmentary segmental   *** Qualifications:** *2U2A and 2U2B: a Proximal 1/3, b Middle 1/3, c Distal 1/3 2U2C: i Proximal diaphyseal-metaphyseal, j Pure diaphyseal, k Distal diaphyseal-metaphyseal* |
|  | **2U3 Distal end segment** | **2U3A Extraarticular**   - 2U3A1 Styloid process - 2U3A2 Simple - 2U3A3 Multifragmentary   **2U3B Partial articular**  **2U3C Complete articular** |
| 7 Hand | **71 Lunate 72 Scaphoid 73 Capitate 74 Hamate 75 Trapezium 76 Other carpal bones 77 Metacarpals 78 Phalanges** | *[Free text]* |
| Notes | *[Free text]* | |
| LOWER LIMB FRACTURE  *(details visible only if “Yes”)* | Yes/No | |
| Location Bone involved | **Bone portion** | **Group** |
| 3 Femur | **31 Proximal end segment** | **31A Trochanteric region**   - 31A1 Simple pertrochanteric - 31A2 Multifragmentary pertrochanteric, lateral wall incompetent (≤20.5 mm) - 31A3 Intertrochanteric (reverse obliquity)   **31B Femoral neck**   - 31B1 Subcapital - 31B2 Transcervical - 31B3 Basicervical   **31C Femoral head**   - 31C1 Split - 31C2 Depression |
|  | **32 Diaphyseal segment** | **32A Simple**   - 32A1* Spiral - 32A2* Oblique (≥30°) - 32A3* Transverse (<30°)   **32B Wedge**   - 32B2* Intact wedge - 32B3* Fragmentary wedge   **32C Multifragmentary**   - 32C2* Intact segmental - 32C3* Fragmentary segmental   *** Qualifications**: *32A and 32B: a Proximal 1/3, b Middle 1/3, c Distal 1/3 32C: i Proximal diaphyseal-metaphyseal, j Pure diaphyseal, k Distal diaphyseal-metaphyseal* |
|  | **33 Distal end segment** | **33A Extraarticular**   - 33A1 Avulsion - 33A2 Simple - 33A3 Wedge or multifragmentary   **33B Partial articular**   - 33B1 Lateral condyle, sagittal - 33B2 Medial condyle, sagittal - 33B3 Frontal (coronal)   **33C Complete articular**   - 33C1 Simple articular, simple metaphyseal - 33C2 Simple articular, wedge or multifragmentary metaphyseal - 33C3 Multifragmentary articular, simple, wedge or multifragmentary metaphyseal |
| 4 Tibia | **41 Proximal end segment** | **41A Extraarticular**   - 41A1 Avulsion - 41A2 Simple - 41A3 Wedge or multifragmentary   **41B Partial articular**   - 41B1 Split - 41B2 Depression - 41B3 Split depression   **41C Complete articular**   - 41C1 Simple articular, simple metaphyseal - 41C2 Simple articular, wedge or multifragmentary metaphyseal - 41C3 Fragmentary or multifragmentary metaphyseal |
|  | **42 Diaphyseal segment** | **42A Simple**   - 42A1* Spiral - 42A2* Oblique (≥30°) - 42A3* Transverse (<30°)   **42B Wedge**   - 42B2* Intact wedge - 42B3* Fragmentary wedge   **42C Multifragmentary**   - 42C2 Intact segmental - 42C3* Fragmentary segmental   *** Qualifications**: *42A and 42B: a Proximal 1/3, b Middle 1/3, c Distal 1/3 42C: i Proximal diaphyseal-metaphyseal, j Pure diaphyseal, k Distal diaphyseal-metaphyseal* |
|  | **43 Distal end segment** | **43A Extraarticular**   - 43A1 Simple - 43A2 Wedge - 43A3 Multifragmentary   **43B Partial articular**   - 43B1 Split - 43B2 Split depression - 43B3 Depression   **43C Complete articular**   - 43C1 Simple articular, simple metaphyseal - 43C2 Simple articular, multifragmentary metaphyseal - 43C3 Multifragmentary articular and multifragmentary metaphyseal |
| 4F Fibula | **4F1 Proximal end segment** | - 4F1A* Simple - 4F1B* Multifragmentary |
|  | **4F2 Diaphyseal segment** | - 4F2A* Simple - 4F2B* Wedge or multifragmentary   *** Qualifications**: *4F1: n Extraarticular, o Intraarticular*  *4F2: a Proximal 1/3, b Middle 1/3, c Distal 1/3* |
|  | **4F3 Distal end segment** | - 4F3A Simple - 4F3B Wedge or multifragmentary |
| 44 Malleolar segment | **44A Infrasyndesmotic fibula injury** | - 44A1 Isolated fibula injury - 44A2 With medial malleolar fracture - 44A3 With posteromedial fracture |
|  | **44B Transsyndesmotic fibula fracture** | - 44B1 Simple fibula fracture - 44B2 With medial injury - 44B3 With medial injury and fracture of the posterolateral rim (Volkmann's fragment) |
|  | **44C Suprasyndesmotic fibula fracture** | - 44C1 Simple diaphyseal fibula fracture - 44C2 Wedge or multifragmentary diaphyseal fibula fracture - 44C3 Proximal fibula injury |
| 8 Foot | **81 Talus 82Calcaneus 83 Navicular 84 Cuboid 85 Cuneiforms 87 Metatarsals 88 Phalanges** | *[Free text]* |
| Notes | *[Free text]* | |
| PERIPHERAL VASCULAR INJURY  *(details visible only if “Yes”)*  Peripheral vascular organ injury scale  **Note: Increase one grade for multiple grade III or IV injuries involving >50% vessel circumference. Decrease one grade for <25% vessel circumference disruption for grades IV or V* | Yes/No | |
|  | ****AAST injury severity scoring 2021 -*** [***https://www.aast.org/resources-detail/injury-scoring-scale***](https://www.aast.org/resources-detail/injury-scoring-scale) | |
|  | Description of injury | - Dissection *[Yes/No]* - Thrombosis *[Yes/No]* - Pseudoaneurysm *[Yes/No]* - Active bleeding *[Yes/No]*   Grade |
|  | - Digital artery/vein - Palmar artery/vein - Deep palmar artery/vein - Dorsalis pedidia artery - Plantar artery/vein - Non-named arterial/venous branches | I |
|  | - Basilic/cephalic vein - Saphenous vein - Radial artery - Ulnar artery | II |
|  | - Axillary vein - Superficial/deep femoral vein - Popliteal vein - Brachial artery - Anterior tibial artery - Posterior tibial artery - Peroneal artery - Tibioperoneal trunk | III |
|  | - Superficial/deep femoral artery - Popliteal artery | IV |
|  | - Axillary artery - Common femoral artery | V |
| Notes | *[Free text]* | |

| OTHER FINDINGS | | |
| --- | --- | --- |
| Fat embolism | Yes/No | If Yes, specify site *[Free text]* |
| Foreign bodies | Yes/No | If Yes, specify site and type *[Free text]* |
| Incidental findings (trauma-unrelated) | *[Free text]* | |

| CONCLUSIONS | *[Free text]* |
| --- | --- |

**IMAGES**

| *FIELD* | *DETAIL* | *ADMITTED VALUES* |
| --- | --- | --- |
| Key images | Key images | *[Images]* |
